# Clinical complete response to serplulimab plus bevacizumab and chemotherapy in MSS/KRAS-mutant metastatic sigmoid colon adenocarcinoma: a case report and literature review

**DOI:** 10.3389/fimmu.2026.1740803

**Published:** 2026-07-01

**Authors:** Qiao Luo, Haiyan Zhang, Binru Di, Yaowen Liu, Yulin Lei, Haixia Liu, Jianmei Wang, Luo Yuhao

**Affiliations:** 1 Department of Oncology, The Affiliated Hospital of Southwest Medical University, Luzhou, China; 2 Department of Pathology, The Affiliated Hospital of Southwest Medical University, Luzhou, China

**Keywords:** mCRC, MSS, immunotherapy, serplulimab, bevacizumab

## Abstract

Mismatched-repair–proficient/microsatellite-stable (pMMR/MSS) metastatic colorectal cancer (mCRC) is typically refractory to immune checkpoint inhibitors. Early randomized data suggest that adding PD-1 blockade to anti-angiogenic therapy and oxaliplatin-based chemotherapy may improve outcomes, but real-world evidence remains limited. A 62-year-old woman presented with advanced sigmoid colon adenocarcinoma and liver, lung, and nodal metastases (cT4aN2bM1b). Molecular profiling showed KRAS p.G12D and pMMR/MSS status. First-line treatment with serplulimab, bevacizumab, and XELOX was initiated. After two cycles, carcinoembryonic antigen (CEA) declined from 3458.2 ng/mL to 1812.84 ng/mL. Over eight induction cycles, cross-sectional imaging demonstrated sustained regression of hepatic and pulmonary metastases without new lesions, representing a near-complete response. Following six cycles of maintenance therapy with serplulimab, bevacizumab, and capecitabine, restaging CT and endoscopy showed no visible tumor, consistent with a clinical complete response (cCR), accompanied by resolution of abdominal pain. The patient remains progression-free beyond 12 months at last follow-up. This case illustrates the feasibility and potential of combining PD-1 blockade, anti-VEGF therapy, and oxaliplatin-based chemotherapy for pMMR/MSS mCRC, aligning with efficacy signals from ASTRUM-015 (phase 2 median PFS 17.2 vs 10.7 months; HR 0.60; MSS subgroup 17.2 vs 10.1 months; HR 0.58). Prospective, biomarker-informed trials are warranted to validate patient selection and durability of benefit.

## Introduction

Colorectal cancer (CRC) is the third most commonly diagnosed cancer and the second leading cause of cancer-related deaths worldwide. In 2022, an estimated 1.9 million new CRC cases and 900,000 deaths were recorded globally, representing about 10% of all cancer incidence and mortality (1). Approximately 20% of patients are diagnosed with *de novo* metastatic disease at presentation, and up to 50% will develop metastases during the disease course (2). Contemporary first-line management of metastatic CRC (mCRC) relies on cytotoxic doublets or triplets such as FOLFOX, FOLFIRI, or FOLFOXIRI combined with targeted agents, including anti-VEGF or anti-EGFR antibodies, according to molecular profile; surgery is generally reserved for symptom control or for resection of limited metastatic deposits in selected candidates. Despite these advances, outcomes remain poor, with a 5-year overall survival (OS) of approximately 15% in mCRC (3).

Approximately 95% of mCRC cases are mismatch repair proficient or microsatellite stable (pMMR/MSS), a subgroup that shows limited responsiveness to single-agent immune checkpoint inhibitors (ICIs). Emerging randomized data suggest that adding PD-1 blockade to anti-VEGF therapy and oxaliplatin-based chemotherapy may confer benefit in this population. In the phase II/III ASTRUM-015 trial (NCT04547166), serplulimab plus bevacizumab biosimilar (HLX04) and XELOX was compared with placebo plus bevacizumab and XELOX as first-line therapy (4). In the phase 2 dataset, independent central review reported a median progression-free survival (PFS) of 17.2 months with serplulimab plus HLX04 and XELOX versus 10.7 months with placebo plus bevacizumab and XELOX, corresponding to a 40% lower risk of progression or death (HR 0.60). In the MSS subgroup, median PFS was 17.2 vs 10.1 months (HR 0.58), and OS data were immature, showing only a trend. Although the ASTRUM-015 trial has reported cCR in pMMR/MSS mCRC, real-world documentation of such deep responses in patients with high tumor burden and multiple metastatic sites remains exceptionally rare. This case adds unique real-world evidence and includes comprehensive longitudinal follow-up, serial imaging, tumor marker kinetics, and baseline immune microenvironment characterization. Therefore, we report a real-world case treated with serplulimab, bevacizumab, and chemotherapy, illustrating the potential depth and durability of response in advanced MSS mCRC when therapy is selected with molecular profiling and multidisciplinary input.

## Case description

**Figure 1:**
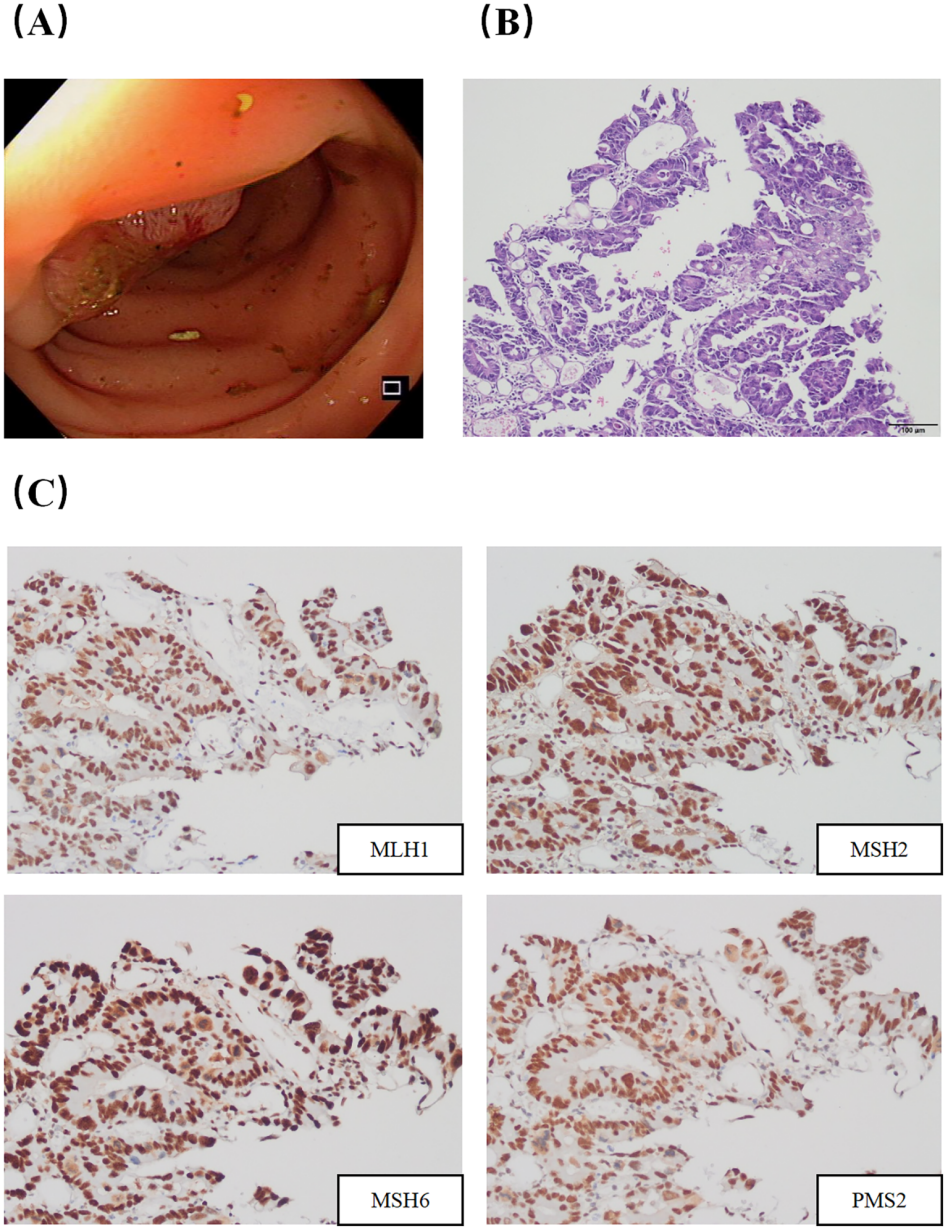
Endoscopic appearance of the primary lesion and pathological analysis of tumor specimens. **(A)** Endoscopic images showing the primary lesion. **(B)** Hematoxylin-eosin (HE) staining of tumor specimens. **(C)** MMR protein immunohistochemical staining.

A 62-year-old woman presented on May 29, 2024, with persistent abdominal pain for more than one month. She reported a one-year history of altered bowel habits and vague upper-abdominal discomfort that had been previously overlooked. Routine screening in April 2024 revealed a markedly elevated carcinoembryonic antigen (CEA, 3629.22 ng/mL), but she deferred further evaluation due to work commitments. On May 29, 2024, contrast-enhanced abdominal CT demonstrated irregular thickening of the sigmoid colon with luminal narrowing consistent with a primary malignancy, multiple hepatic metastases, bilateral pulmonary metastases, and nodal metastases involving the para-aortic, bilateral iliac, and mesenteric stations. Colonoscopy with biopsy on June 6, 2024 confirmed sigmoid colon adenocarcinoma (**Figures 1A, B**). Molecular profiling revealed a KRAS p.G12D mutation with MSS phenotype. Further molecular profiling revealed a tumor mutational burden (TMB) of 4.2 mut/Mb (low). No pathogenic germline mutations were detected in POLE, POLD1, or mismatch repair genes. The patient denied any family history of colorectal cancer or Lynch syndrome-associated malignancies. Immunohistochemistry demonstrated intact expression of MLH1, MSH2, MSH6, and PMS2, thereby ruling out Lynch syndrome (**Figure 1C**). The final diagnosis was sigmoid colon adenocarcinoma with hepatic, pulmonary, and nodal metastases, clinical stage cT4aN2bM1b (AJCC 8th edition), corresponding to stage IVB.

**Figure 2:**
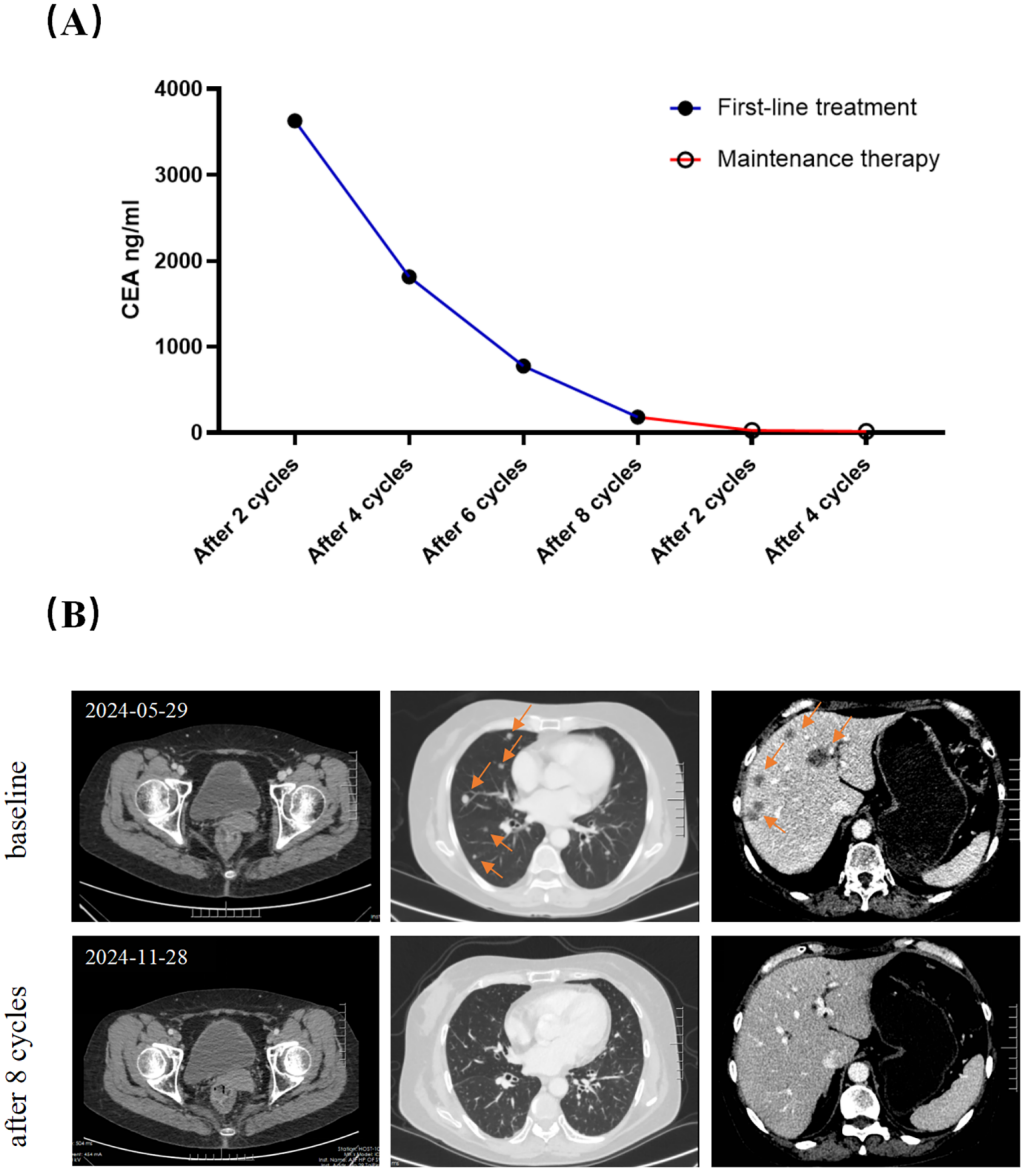
Longitudinal changes in CEA levels and imaging evaluation of lesions during treatment. **(A)** The CEA level demonstrated a consistent decline throughout the course of treatment. **(B)** CT imaging changes of primary lesions, liver and lung metastases before and after treatment.

**Figure 3:**
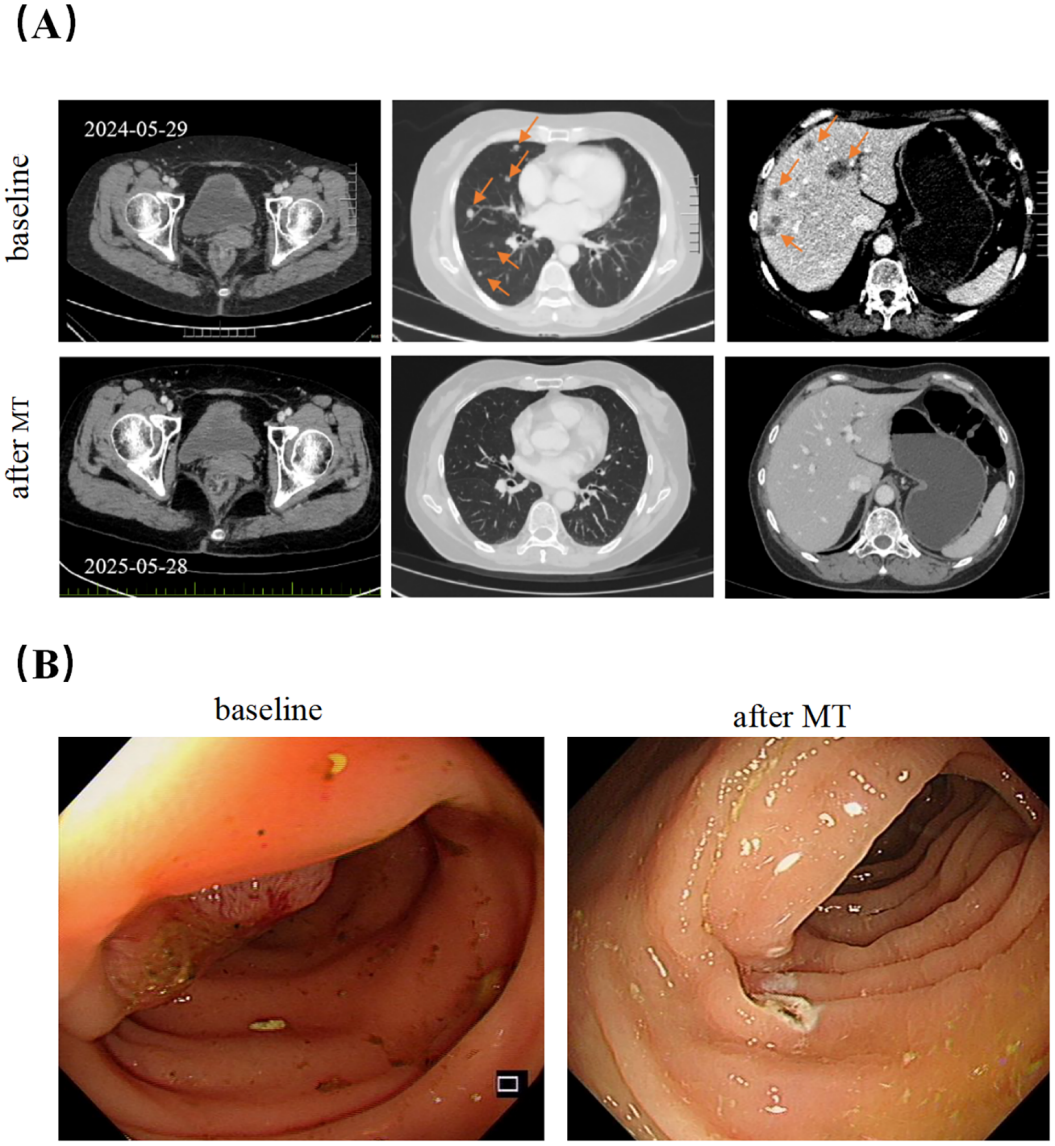
Radiographic and endoscopic comparison of primary and metastatic lesions before and after maintenance therapy. **(A)** CT imaging changes of primary lesions, liver and lung metastases before treatment and after maintenance therapy. **(B)** Comparison of endoscopic images before and after maintenance therapy.

**Figure 4:**
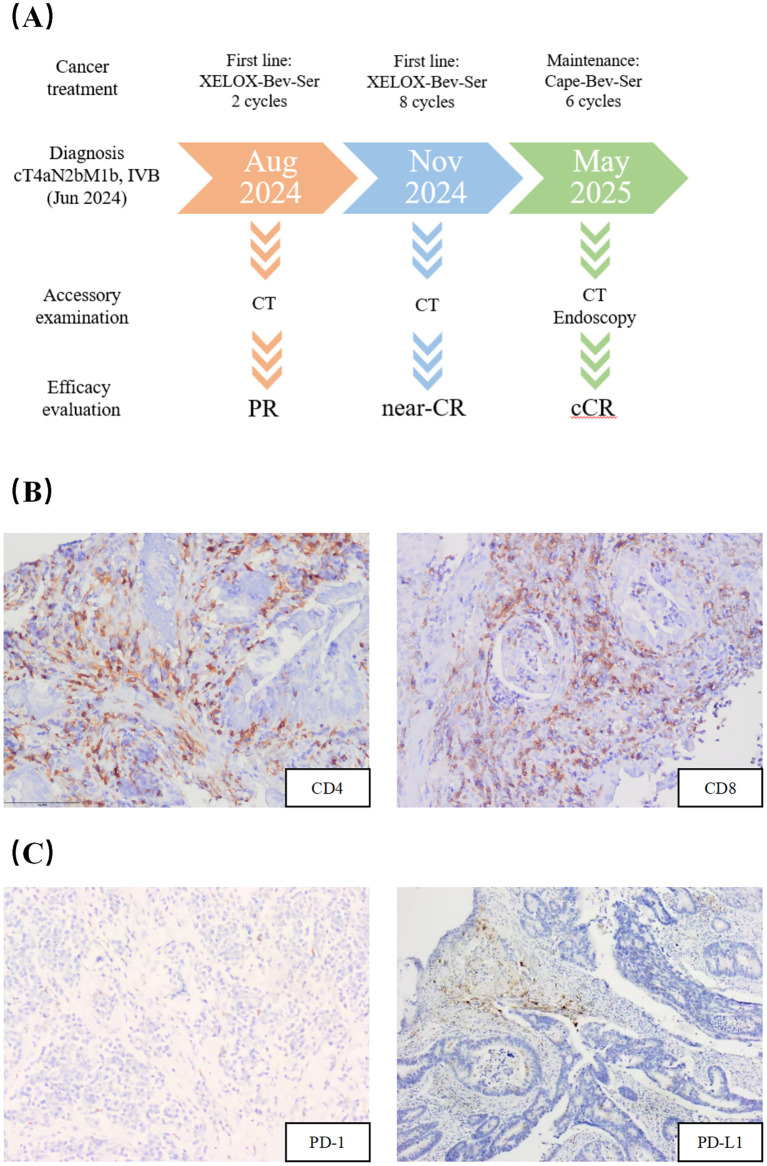
Treatment timeline and lymphocyte infiltration in TME. **(A)** Timeline of auxiliary examinations and efficacy evaluation during treatment. **(B)** Infiltration of CD4^+^ and CD8^+^ T lymphocytes within the TME. **(C)** Immunohistochemical staining of PD-1 and PD-L1.

Given the molecular profile (*KRAS* p.G12D, MSS) and the absence of bowel obstruction, the multidisciplinary team (MDT) recommended systemic therapy. On June 14, 2024, the patient initiated serplulimab, bevacizumab, and XELOX chemotherapy, informed by emerging evidence from the ASTRUM-015 trial. After two cycles, CEA decreased from 3458.2 ng/mL (June 14, 2024) to 1812.84 ng/mL (August 14, 2024) (**Figure 2A**). Over the subsequent six months, the patient completed eight cycles of serplulimab, bevacizumab, and XELOX. Serial imaging showed progressive reduction of liver and lung lesions with no new lesions. The sum of diameters of target lesions (liver and lung) decreased by 97%, corresponding to a near-complete response, accompanied by a sustained decline in CEA levels (**Figures 2A, B**). After an additional six cycles of maintenance therapy (serplulimab, bevacizumab, and capecitabine), CT and endoscopic re-examination demonstrated complete tumor disappearance, and abdominal pain resolved completely, meeting criteria for clinical complete response (cCR) (**Figures 3A, B**). Throughout the treatment course, no grade ≥3 adverse events were observed. Grade 1 hand-foot syndrome occurred after cycle 2 and was managed symptomatically. No immune-related enteritis, hepatitis, pneumonitis, or myocarditis was reported. Following the aforementioned treatment (**Figure 4A**), the patient reported a marked improvement in quality of life, with complete resolution of abdominal pain, regained physical strength, and the ability to perform routine daily activities. The patient expressed high satisfaction with the therapeutic outcome and has remained compliant with ongoing follow-up and management. Presently, the patient remains on active treatment with serplulimab-based maintenance therapy in our department. Continued follow-up is being conducted to assess long-term outcomes and treatment tolerance.

## Discussion

Despite decades of incremental advances, conventional chemotherapy and targeted therapy for mCRC remain constrained by limited durability and biomarker-defined eligibility. Chemotherapy regimens such as FOLFOX or XELOX provide initial disease control, but their efficacy is transient, with median PFS rarely exceeding 10 months in first-line setting (5). Anti-EGFR antibodies (cetuximab or panitumumab) are not recommended for tumors harboring *RAS* mutations, which occur in about 40 to 50% of mCRC, because such mutations predict lack of benefit from EGFR blockade (2, 6). By contrast, VEGF inhibition with bevacizumab offers modest survival gains whose magnitude depends on the chemotherapy backbone: in AVF2107g (7), adding bevacizumab to irinotecan-based therapy improved median OS by 4.7 months (20.3 vs 15.6 months), whereas in the oxaliplatin-based NO16966 program, pooled analyses showed smaller and in some analyses non-significant differences, on the order of 1.4 to approximately 2 months (8).

Immunotherapy has revolutionized outcomes for the microsatellite instability-high (MSI-H) or mismatch repair-deficient (dMMR) subset of mCRC (9, 10). The phase III KEYNOTE-177 trial (NCT02563002) demonstrated that pembrolizumab (anti-PD-1) significantly improved PFS versus chemotherapy (mPFS: 16.5 vs. 8.2 months; HR = 0.59), with fewer grade ≥3 adverse events; and 3-year OS rates were 61.4% vs. 50.3% in the pembrolizumab and chemotherapy arms, respectively (11). In the phase 2 CheckMate-142 trial (NCT02060188), nivolumab plus low-dose ipilimumab achieved an objective response rate (ORR) of 69% with a median duration of response not reached and 13% complete responses at extended follow-up (12).

Regrettably, ICIs have not reproduced in pMMR/MSS CRC the success observed in the dMMR/MSI-H subtype. The prevailing view attributes this divergence to immunologic “hot versus cold” phenotypes. The pMMR/MSS tumors represent immunologically “cold”, characterized by low tumor mutational burden (TMB <5 mut/Mb), limited neoantigen generation that blunts dendritic cell priming, and downregulated MHC-I expression further constrains CD8^+^ T-cell recognition (13, 14). In addition, the tumor microenvironment (TME) is immunosuppressive, with enrichment of regulatory T cells (Tregs) and myeloid-derived suppressor cells (MDSCs) that release IL-10 and TGF-β, along with VEGF-driven abnormal angiogenesis that creates physical and functional barriers to T-cell infiltration (15). These mechanisms provide a biologic rationale for combining PD-1 blockade with anti-angiogenic therapy and cytotoxic chemotherapy to convert immune-excluded tumors into more permissive microenvironments (16).

Meanwhile, numerous clinical trials have consistently demonstrated that single-agent PD-1/PD-L1 blockade has minimal activity in pMMR/MSS mCRC. In the seminal phase 2 study by Le and colleagues, pembrolizumab monotherapy demonstrated an immune-related ORR of 0% and an immune-related 6-month PFS rate of 11% in pMMR/MSS mCRC, in sharp contrast to responses in dMMR/MSI-H disease (17). Outside CRC, first-line pembrolizumab monotherapy was not superior to chemotherapy for OS or PFS in the phase 3 KEYNOTE-062 trial of advanced gastric/gastroesophageal junction cancer, underscoring that checkpoint monotherapy does not reliably outperform cytotoxic therapy in MSS non-T-cell-inflamed tumors (18). Overwise, the large randomized evidence of IMblaze370 trial in chemorefractory MSS mCRC has likewise been negative (19). Atezolizumab (either alone or combined with the MEK inhibitor cobimetinib) did not improve OS versus regorafenib, underscoring that reversing immune exclusion in MSS disease will require alternative, mechanism-based combination strategies.

Overcoming immune exclusion in pMMR/MSS mCRC likely requires mechanistically synergistic combinations. Cytotoxic chemotherapy can remodel the TME by depleting immunosuppressive cells, enhancing immune infiltration and NK-cell activity, and inducing immunogenic cell death (ICD) that promotes dendritic-cell priming (20). Anti-angiogenic therapy such as bevacizumab can normalize vasculature, improve T-cell trafficking, and mitigate Treg/MDSC-mediated suppression, providing a biologic rationale for pairing PD-1/PD-L1 blockade with VEGF inhibition and chemotherapy (21). Early clinical signals are consistent with this rationale. In the phase II AtezoTRIBE trial, adding atezolizumab to first-line FOLFOXIRI plus bevacizumab extended mPFS in the overall population, with a 1.5-month mPFS improvement observed in the pMMR subgroup (22). In the randomized phase 2 portion of ASTRUM-015, serplulimab plus HLX04 (a bevacizumab biosimilar) and XELOX achieved a median PFS of 17.2 months versus 10.7 months with placebo plus bevacizumab and XELOX (HR 0.60, a 40% lower risk of progression/death); in the MSS subgroup, median PFS was 17.2 vs 10.1 months (HR 0.58). Similarly, the BBCAPX single-arm study in *RAS*-mutant, MSS mCRC reported an ORR of 84.0% and a DCR of 100% with sintilimab plus CapeOX and bevacizumab (23).

This case underscores two clinically relevant points. First, combining PD-1 blockade with anti-angiogenic therapy and cytotoxic chemotherapy is feasible in pMMR/MSS mCRC, a subtype historically refractory to immunotherapy. Second, the regimen produced a deep and durable antitumor effect: radiologic near-CR was achieved within eight cycles, and subsequent maintenance therapy consolidated the response to a cCR, accompanied by resolution of cancer-related symptoms and preserved functional status. The combination regimen containing Serplulimab, together with chemotherapy and bevacizumab, may have contributed to the deep response; however, the relative contribution of each agent cannot be determined from a single case. Although cross-trial comparisons are inappropriate for a single case, the observed depth of response and progression-free interval exceeding 12 months appear favorable relative to typical outcomes reported with chemotherapy plus targeted therapy in similar populations. These findings are hypothesis generating and align with emerging efficacy signals from the ASTRUM-015 trial. Larger, biomarker-informed triplet strategies in prospective studies should further test this approach and may expand the therapeutic benefit for patients with pMMR/MSS mCRC.

We postulate that the profound therapeutic response observed in this case can be attributed to a high degree of pre-existing immune cell infiltration within the TME (24). To validate this hypothesis, we performed a retrospective immunohistochemical staining analysis on the patient’s pre-treatment tumor tissue specimen. The results unequivocally demonstrated a substantial infiltration of both CD4^+^ and CD8^+^ T lymphocytes within the TME prior to therapy (**Figure 4B**). This finding suggests that the characteristics of the baseline tumor immune microenvironment—particularly the density and spatial distribution of pre-existing CD8^+^ cytotoxic T lymphocytes and CD4^+^ helper T cells—may serve as a critical predictive biomarker for patient benefit from PD-1 inhibitor-based combination therapies in pMMR/MSS mCRC. In contrast to “immune-desert” or “immune-excluded” phenotypes, it is plausible that in MSS subtypes that are basally “immune-inflamed,” the pre-existing anti-tumor immune foundation can be more readily reactivated and synergistically enhanced by the combination therapy, thereby eliciting a deep and durable response.

In addition, immunohistochemistry (IHC) for PD-L1 and PD-1 was performed on the pre-treatment tumor tissue specimen (**Figure 4C**). Both PD-L1 expression on tumor cells (CPS < 1) and PD-1 expression on tumor-infiltrating lymphocytes were negative. These negative findings suggest that the deep response observed in this case may not be attributed to high baseline PD-L1 expression or PD-1^+^ T-cell enrichment, but rather to other immune microenvironment features, such as pre-existing CD4^+^ and CD8^+^ T-cell infiltration.

Furthermore, the patient in this case harbored a KRAS G12D mutation, a molecular alteration that has been increasingly recognized as an active driver of an immunosuppressive tumor microenvironment (25, 26). Against this immunosuppressive backdrop, the profound and durable therapeutic response observed in this case appears counterintuitive. However, the concurrent administration of oxaliplatin-based chemotherapy (XELOX regimen) and the anti-VEGF antibody bevacizumab likely remodeled the tumor microenvironment in a manner that partially overcomes KRAS G12D-driven immune suppression. The substantial baseline infiltration of CD4^+^ and CD8^+^ T cells documented in this case may have provided a foundation for synergistic interaction with these treatment-induced immunomodulatory effects. Thus, despite the immunosuppressive pressure exerted by the KRAS G12D mutation, the combination of chemotherapy and anti-angiogenic therapy may effectively “re-educate” the microenvironment, converting a resistant phenotype into one that permits a deep and sustained response to PD-1 blockade.

However, it should be noted that the prevalence of such baseline immune features in pMMR/MSS colorectal cancer remains limited. A study comparing the immune landscape at the tumor invasion front reported that the proportion of CD8^+^ T cells in pMMR/MSS colorectal cancer was only 6.29% ± 1.62%, significantly lower than the 26.84% ± 3.17% observed in dMMR colorectal cancer (p < 0.001) (27). This indicates that only a small subset of pMMR/MSS tumors exhibit detectable T-cell infiltration at baseline. More importantly, the predictive value of pre-existing T-cell infiltration for response to immune checkpoint inhibitors in this population remains uncertain and requires validation through prospective, biomarker-informed studies.

A major strength of this case is the availability of longitudinal, multimodal follow-up that includes serial imaging, endoscopic documentation, and tumor marker kinetics before, during, and after treatment. High-resolution contrast-enhanced CT obtained at critical timepoints—baseline in May 2024, after 8 cycles in November 2024, and during the maintenance phase—demonstrates marked regression of hepatic and pulmonary metastases with resolution of nodal enlargement. Flexible sigmoidoscopy with photographic documentation demonstrates interval reduction of the primary sigmoid lesion and restoration of near-normal luminal patency; on reassessment during maintenance, the mucosal surface appeared normal. These objective findings align with a sustained decline in serum CEA from 3458.20 ng/mL to 15.74 ng/mL. Taken together, the consistent improvements across imaging, endoscopy, and biomarkers provide clear real-world documentation of treatment effect in this pMMR/MSS setting. Furthermore, since clinical complete response does not equate to pathological complete response and CT may miss minimal or metabolically active residual disease, we plan to perform whole-body PET/CT at 18 months after treatment initiation to detect possible occult residual lesions and guide whether to continue or de-escalate subsequent therapy.

Finally, this case offers an important practical lesson. Blood-based biomarkers such as methylated ctDNA, cell-free RNA, and CEA have been shown to identify colorectal cancer prior to metastasis (28). However, the present case demonstrates that even a markedly elevated screening CEA (3629.22 ng/mL) may not translate into early diagnosis if real-world barriers intervene – here, the patient delayed further evaluation due to work-related obligations, presenting with stage IVB disease. Therefore, the successful implementation of early detection requires not only sensitive biomarkers but also timely diagnostic follow-up. Healthcare systems and patients alike should prioritize the evaluation of abnormal blood-based markers, irrespective of competing personal commitments.

## Data Availability

The original contributions presented in the study are included in the article/supplementary material. Further inquiries can be directed to the corresponding authors.
